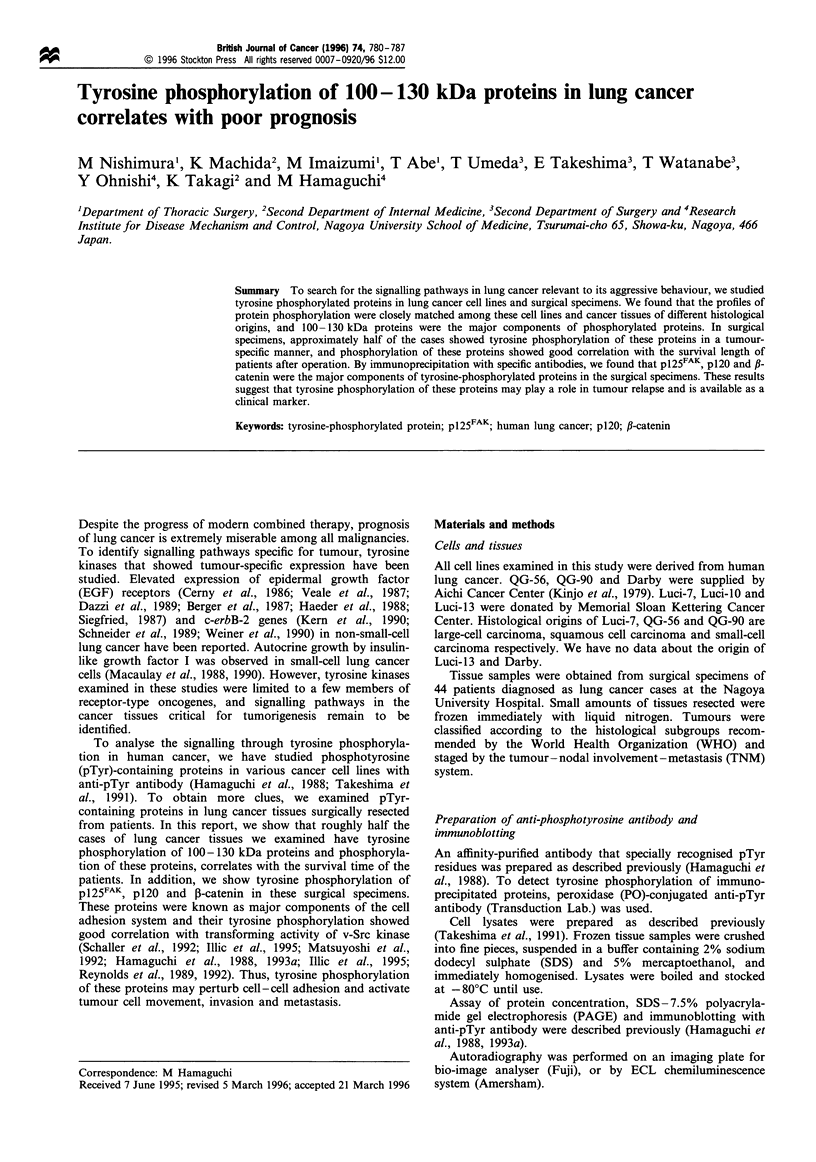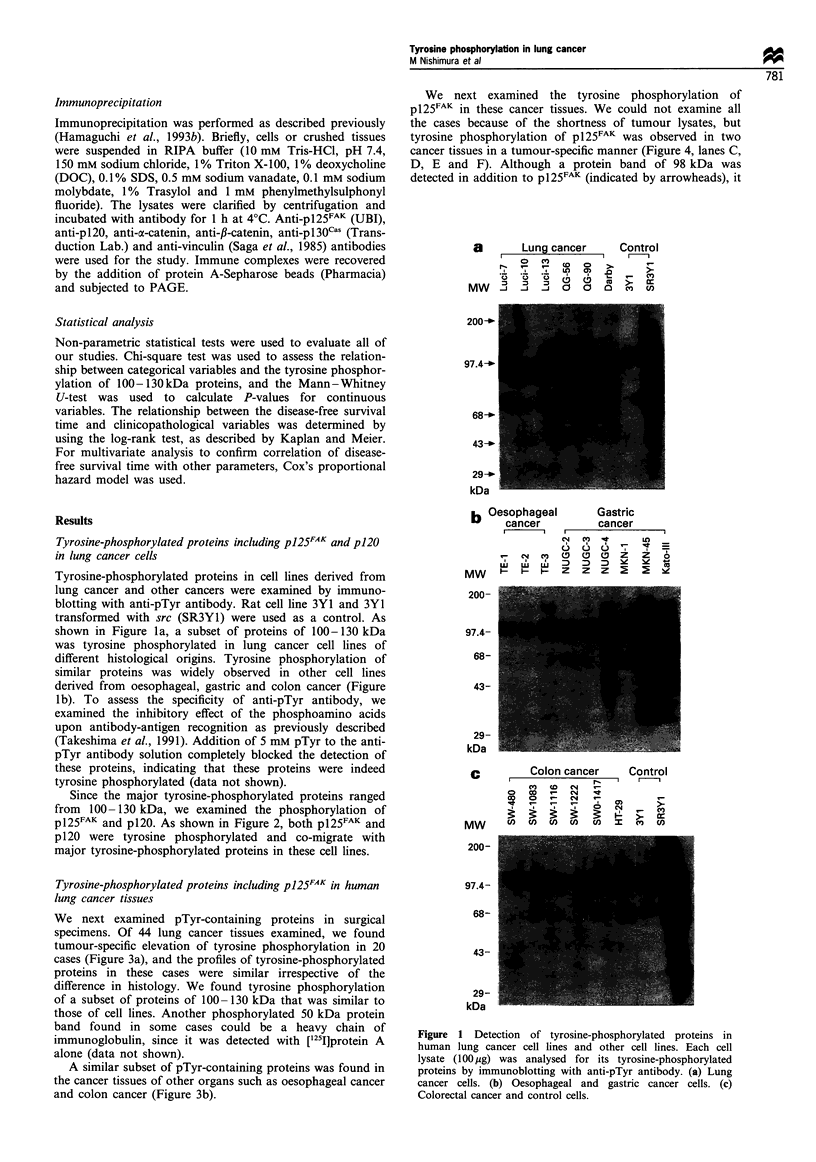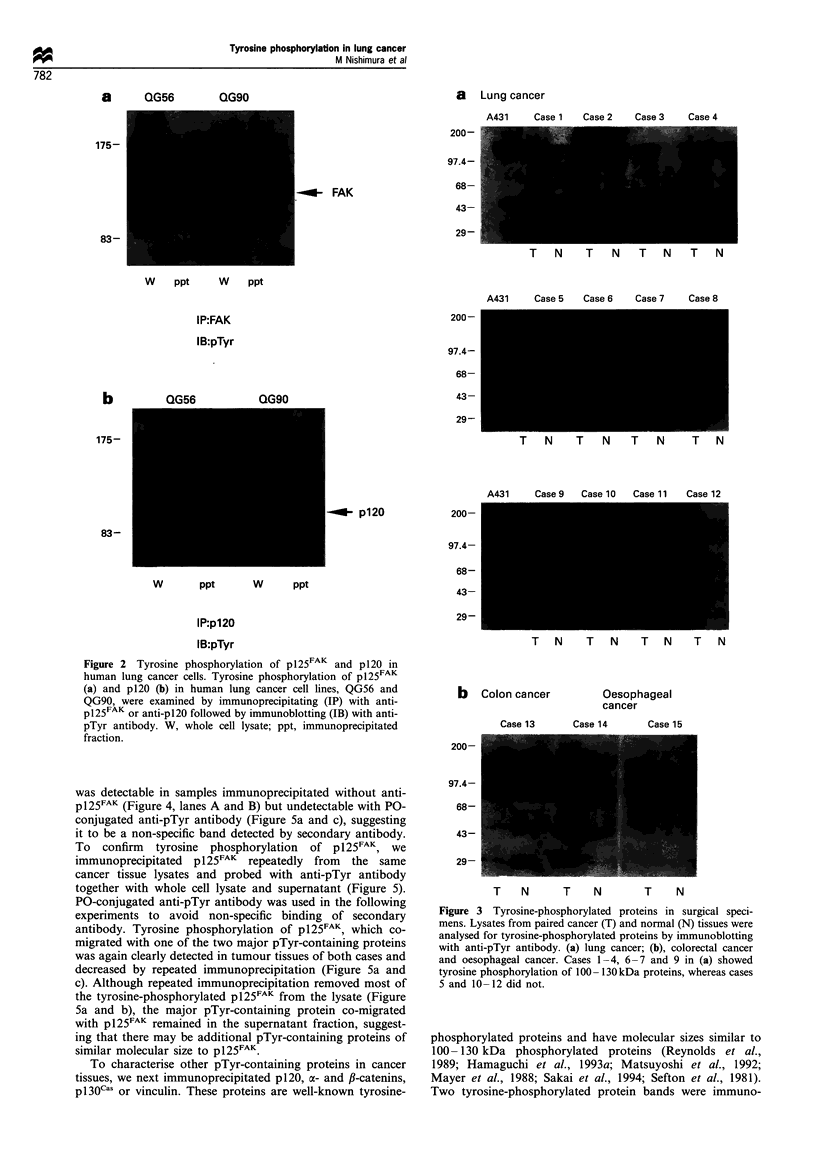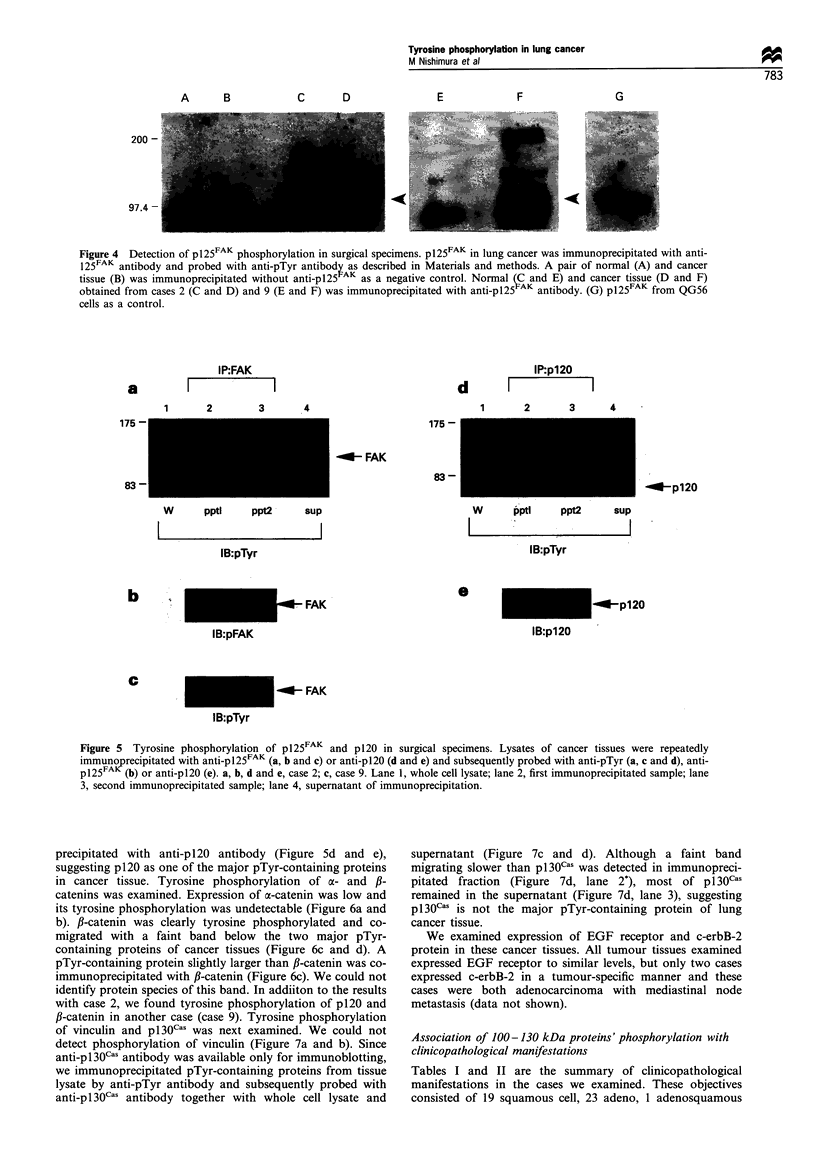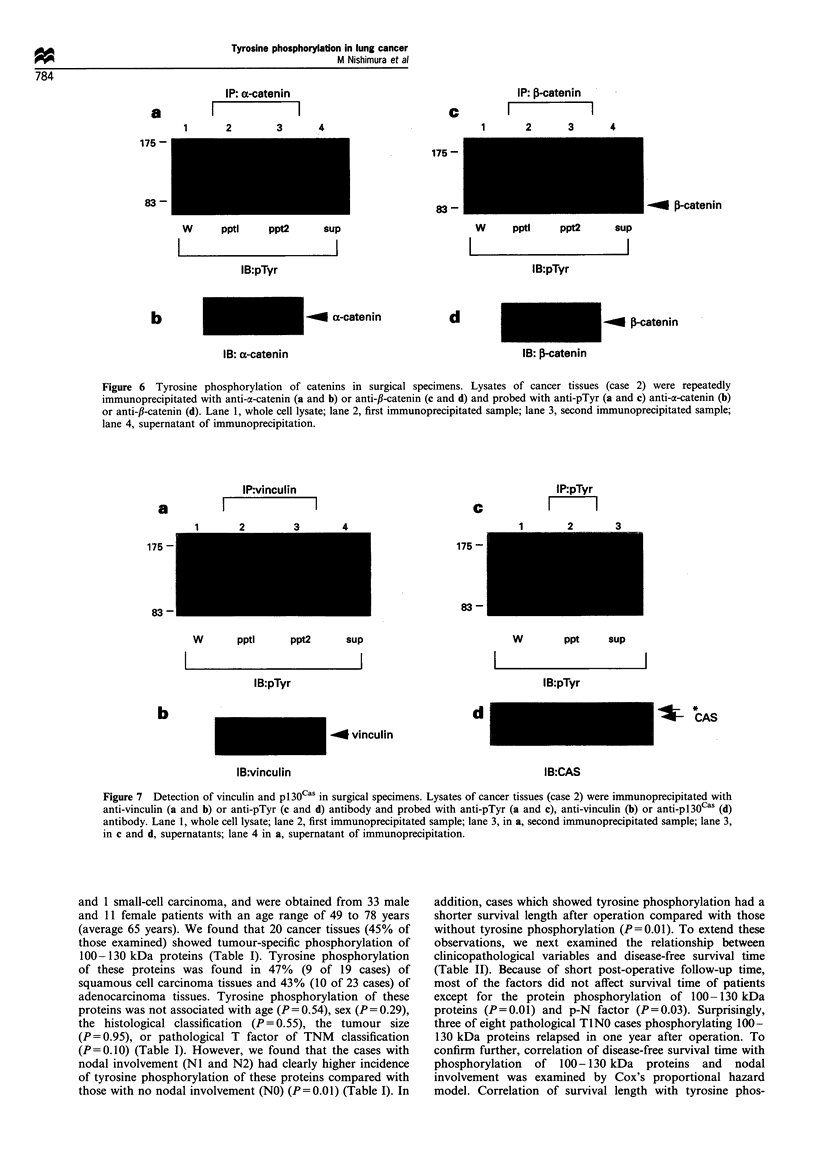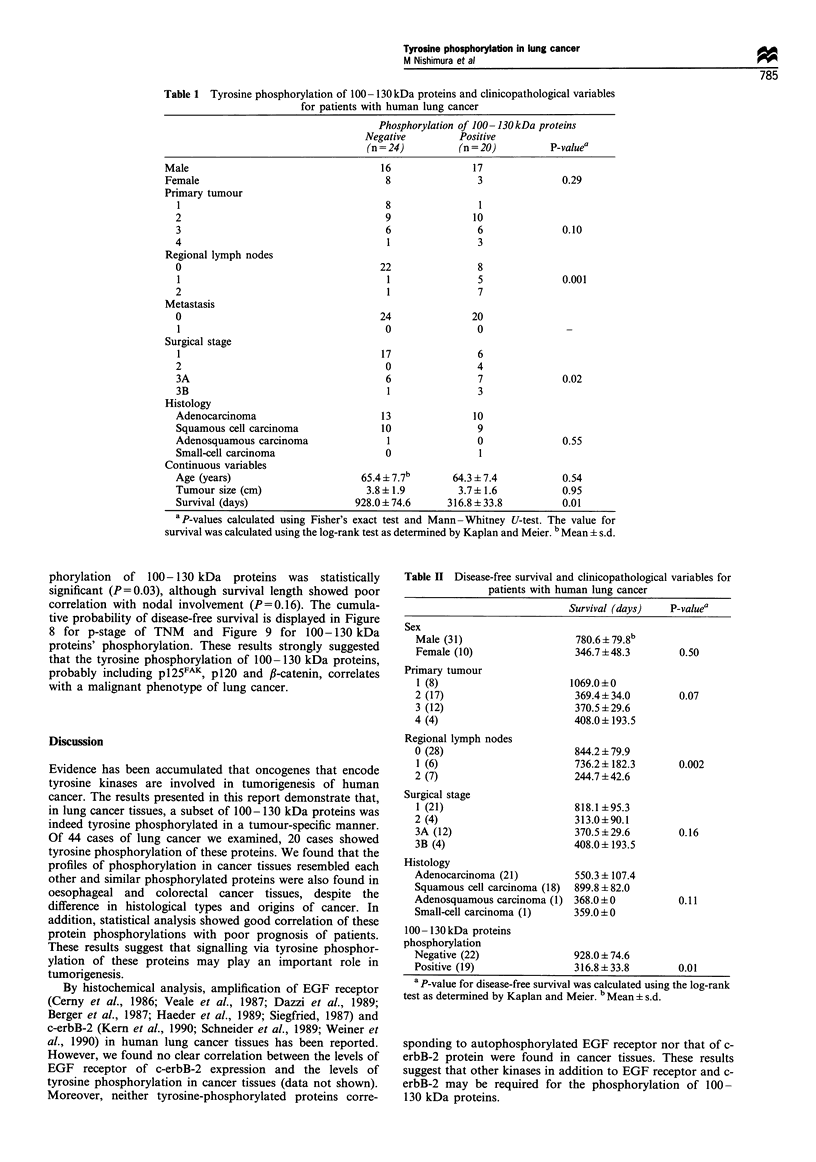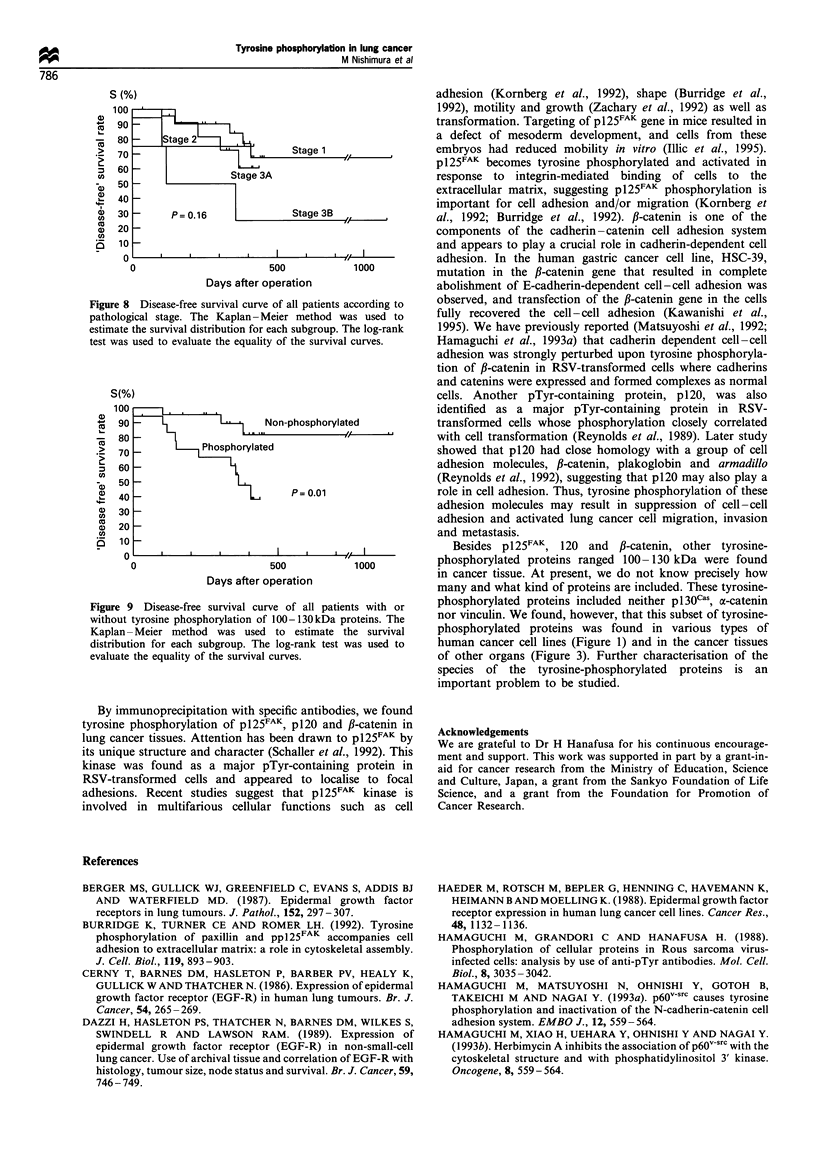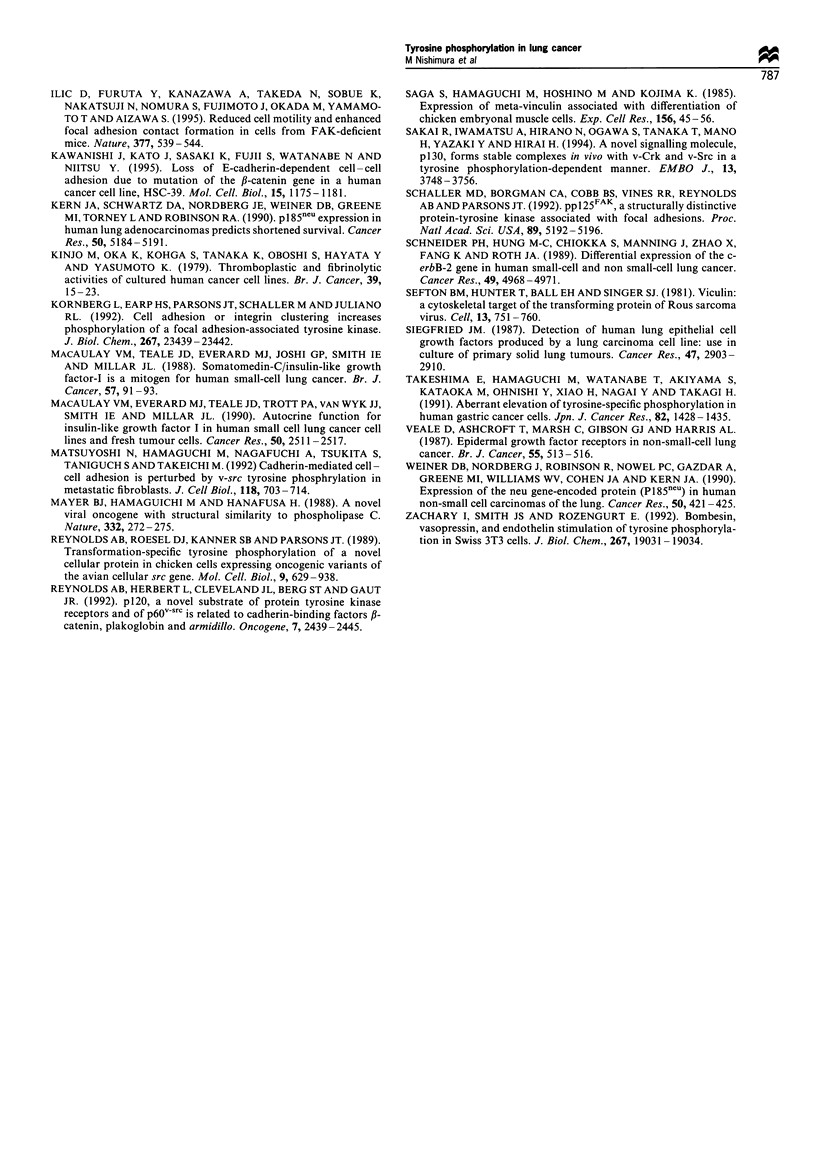# Tyrosine phosphorylation of 100-130 kDa proteins in lung cancer correlates with poor prognosis.

**DOI:** 10.1038/bjc.1996.436

**Published:** 1996-09

**Authors:** M. Nishimura, K. Machida, M. Imaizumi, T. Abe, T. Umeda, E. Takeshima, T. Watanabe, Y. Ohnishi, K. Takagi, M. Hamaguchi

**Affiliations:** Department of Thoracic Surgery, Nagoya University School of Medicine, Japan.

## Abstract

**Images:**


British Journal of Cancer (1996) 74, 780-787
© 1996 Stockton Press All rights reserved 0007-0920/96 $12.00

### Tyrosine phosphorylation of 100-130 kDa proteins in lung cancer correlates with poor prognosis

M Nishimura[1], K Machida[2], M Imaizumi[1], T Abe[1], T Umeda[3], E Takeshima[3], T Watanabe[3], Y Ohnishi[4], K Takagi[2] and M Hamaguchi[4]

[1]*Department of Thoracic Surgery,* [2]*Second Department of Internal Medicine,* [3]*Second Department of Surgery and* [4]*Research Institute for Disease Mechanism and Control, Nagoya University School of Medicine, Tsurumai-cho 65, Showa-ku, Nagoya, 466 Japan.*

**Summary** To search for the signalling pathways in lung cancer relevant to its aggressive behaviour, we studied tyrosine phosphorylated proteins in lung cancer cell lines and surgical specimens. We found that the profiles of protein phosphorylation were closely matched among these cell lines and cancer tissues of different histological origins, and 100-130 kDa proteins were the major components of phosphorylated proteins. In surgical specimens, approximately half of the cases showed tyrosine phosphorylation of these proteins in a tumour-specific manner, and phosphorylation of these proteins showed good correlation with the survival length of patients after operation. By immunoprecipitation with specific antibodies, we found that $p125^{FAK}$, p120 and β-catenin were the major components of tyrosine-phosphorylated proteins in the surgical specimens. These results suggest that tyrosine phosphorylation of these proteins may play a role in tumour relapse and is available as a clinical marker.

**Keywords:** tyrosine-phosphorylated protein; $p125^{FAK}$; human lung cancer; p120; β-catenin

Despite the progress of modern combined therapy, prognosis of lung cancer is extremely miserable among all malignancies. To identify signalling pathways specific for tumour, tyrosine kinases that showed tumour-specific expression have been studied. Elevated expression of epidermal growth factor (EGF) receptors (Cerny *et al.*, 1986; Veale *et al.*, 1987; Dazzi *et al.*, 1989; Berger *et al.*, 1987; Haeder *et al.*, 1988; Siegfried, 1987) and c-*erb*B-2 genes (Kern *et al.*, 1990; Schneider *et al.*, 1989; Weiner *et al.*, 1990) in non-small-cell lung cancer have been reported. Autocrine growth by insulin-like growth factor I was observed in small-cell lung cancer cells (Macaulay *et al.*, 1988, 1990). However, tyrosine kinases examined in these studies were limited to a few members of receptor-type oncogenes, and signalling pathways in the cancer tissues critical for tumorigenesis remain to be identified.

To analyse the signalling through tyrosine phosphorylation in human cancer, we have studied phosphotyrosine (pTyr)-containing proteins in various cancer cell lines with anti-pTyr antibody (Hamaguchi *et al.*, 1988; Takeshima *et al.*, 1991). To obtain more clues, we examined pTyr-containing proteins in lung cancer tissues surgically resected from patients. In this report, we show that roughly half the cases of lung cancer tissues we examined have tyrosine phosphorylation of 100-130 kDa proteins and phosphorylation of these proteins, correlates with the survival time of the patients. In addition, we show tyrosine phosphorylation of $p125^{FAK}$, p120 and β-catenin in these surgical specimens. These proteins were known as major components of the cell adhesion system and their tyrosine phosphorylation showed good correlation with transforming activity of v-Src kinase (Schaller *et al.*, 1992; Illic *et al.*, 1995; Matsuyoshi *et al.*, 1992; Hamaguchi *et al.*, 1988, 1993*a*; Illic *et al.*, 1995; Reynolds *et al.*, 1989, 1992). Thus, tyrosine phosphorylation of these proteins may perturb cell-cell adhesion and activate tumour cell movement, invasion and metastasis.

Correspondence: M Hamaguchi
Received 7 June 1995; revised 5 March 1996; accepted 21 March 1996

### Materials and methods

### *Cells and tissues*

All cell lines examined in this study were derived from human lung cancer. QG-56, QG-90 and Darby were supplied by Aichi Cancer Center (Kinjo *et al.*, 1979). Luci-7, Luci-10 and Luci-13 were donated by Memorial Sloan Kettering Cancer Center. Histological origins of Luci-7, QG-56 and QG-90 are large-cell carcinoma, squamous cell carcinoma and small-cell carcinoma respectively. We have no data about the origin of Luci-13 and Darby.

Tissue samples were obtained from surgical specimens of 44 patients diagnosed as lung cancer cases at the Nagoya University Hospital. Small amounts of tissues resected were frozen immediately with liquid nitrogen. Tumours were classified according to the histological subgroups recommended by the World Health Organization (WHO) and staged by the tumour-nodal involvement-metastasis (TNM) system.

### *Preparation of anti-phosphotyrosine antibody and immunoblotting*

An affinity-purified antibody that specially recognised pTyr residues was prepared as described previously (Hamaguchi *et al.*, 1988). To detect tyrosine phosphorylation of immunoprecipitated proteins, peroxidase (PO)-conjugated anti-pTyr antibody (Transduction Lab.) was used.

Cell lysates were prepared as described previously (Takeshima *et al.*, 1991). Frozen tissue samples were crushed into fine pieces, suspended in a buffer containing 2% sodium dodecyl sulphate (SDS) and 5% mercaptoethanol, and immediately homogenised. Lysates were boiled and stocked at −80°C until use.

Assay of protein concentration, SDS-7.5% polyacrylamide gel electrophoresis (PAGE) and immunoblotting with anti-pTyr antibody were described previously (Hamaguchi *et al.*, 1988, 1993*a*).

Autoradiography was performed on an imaging plate for bio-image analyser (Fuji), or by ECL chemiluminescence system (Amersham).

*Immunoprecipitation*

Immunoprecipitation was performed as described previously (Hamaguchi *et al.*, 1993*b*). Briefly, cells or crushed tissues were suspended in RIPA buffer (10 mM Tris-HCl, pH 7.4, 150 mM sodium chloride, 1% Triton X-100, 1% deoxycholine (DOC), 0.1% SDS, 0.5 mM sodium vanadate, 0.1 mM sodium molybdate, 1% Trasylol and 1 mM phenylmethylsulphonyl fluoride). The lysates were clarified by centrifugation and incubated with antibody for 1 h at 4°C. Anti-p125$^{FAK}$ (UBI), anti-p120, anti-$\alpha$-catenin, anti-$\beta$-catenin, anti-p130$^{Cas}$ (Transduction Lab.) and anti-vinculin (Saga *et al.*, 1985) antibodies were used for the study. Immune complexes were recovered by the addition of protein A-Sepharose beads (Pharmacia) and subjected to PAGE.

*Statistical analysis*

Non-parametric statistical tests were used to evaluate all of our studies. Chi-square test was used to assess the relationship between categorical variables and the tyrosine phosphorylation of 100–130 kDa proteins, and the Mann–Whitney *U*-test was used to calculate *P*-values for continuous variables. The relationship between the disease-free survival time and clinicopathological variables was determined by using the log-rank test, as described by Kaplan and Meier. For multivariate analysis to confirm correlation of disease-free survival time with other parameters, Cox's proportional hazard model was used.

### Results

*Tyrosine-phosphorylated proteins including p125$^{FAK}$ and p120 in lung cancer cells*

Tyrosine-phosphorylated proteins in cell lines derived from lung cancer and other cancers were examined by immunoblotting with anti-pTyr antibody. Rat cell line 3Y1 and 3Y1 transformed with *src* (SR3Y1) were used as a control. As shown in Figure 1a, a subset of proteins of 100–130 kDa was tyrosine phosphorylated in lung cancer cell lines of different histological origins. Tyrosine phosphorylation of similar proteins was widely observed in other cell lines derived from oesophageal, gastric and colon cancer (Figure 1b). To assess the specificity of anti-pTyr antibody, we examined the inhibitory effect of the phosphoamino acids upon antibody-antigen recognition as previously described (Takeshima *et al.*, 1991). Addition of 5 mM pTyr to the anti-pTyr antibody solution completely blocked the detection of these proteins, indicating that these proteins were indeed tyrosine phosphorylated (data not shown).

Since the major tyrosine-phosphorylated proteins ranged from 100–130 kDa, we examined the phosphorylation of p125$^{FAK}$ and p120. As shown in Figure 2, both p125$^{FAK}$ and p120 were tyrosine phosphorylated and co-migrate with major tyrosine-phosphorylated proteins in these cell lines.

*Tyrosine-phosphorylated proteins including p125$^{FAK}$ in human lung cancer tissues*

We next examined pTyr-containing proteins in surgical specimens. Of 44 lung cancer tissues examined, we found tumour-specific elevation of tyrosine phosphorylation in 20 cases (Figure 3a), and the profiles of tyrosine-phosphorylated proteins in these cases were similar irrespective of the difference in histology. We found tyrosine phosphorylation of a subset of proteins of 100–130 kDa that was similar to those of cell lines. Another phosphorylated 50 kDa protein band found in some cases could be a heavy chain of immunoglobulin, since it was detected with [$^{125}$I]protein A alone (data not shown).

A similar subset of pTyr-containing proteins was found in the cancer tissues of other organs such as oesophageal cancer and colon cancer (Figure 3b).

We next examined the tyrosine phosphorylation of p125$^{FAK}$ in these cancer tissues. We could not examine all the cases because of the shortness of tumour lysates, but tyrosine phosphorylation of p125$^{FAK}$ was observed in two cancer tissues in a tumour-specific manner (Figure 4, lanes C, D, E and F). Although a protein band of 98 kDa was detected in addition to p125$^{FAK}$ (indicated by arrowheads), it

**Figure 1** Detection of tyrosine-phosphorylated proteins in human lung cancer cell lines and other cell lines. Each cell lysate (100 $\mu$g) was analysed for its tyrosine-phosphorylated proteins by immunoblotting with anti-pTyr antibody. (**a**) Lung cancer cells. (**b**) Oesophageal and gastric cancer cells. (**c**) Colorectal cancer and control cells.

**Figure 2** Tyrosine phosphorylation of $p125^{FAK}$ and p120 in human lung cancer cells. Tyrosine phosphorylation of $p125^{FAK}$ (**a**) and p120 (**b**) in human lung cancer cell lines, QG56 and QG90, were examined by immunoprecipitating (IP) with anti-$p125^{FAK}$ or anti-p120 followed by immunoblotting (IB) with anti-pTyr antibody. W, whole cell lysate; ppt, immunoprecipitated fraction.

**Figure 3** Tyrosine-phosphorylated proteins in surgical specimens. Lysates from paired cancer (T) and normal (N) tissues were analysed for tyrosine-phosphorylated proteins by immunoblotting with anti-pTyr antibody. (**a**) lung cancer; (**b**), colorectal cancer and oesophageal cancer. Cases 1–4, 6–7 and 9 in (**a**) showed tyrosine phosphorylation of 100–130 kDa proteins, whereas cases 5 and 10–12 did not.

was detectable in samples immunoprecipitated without anti-$p125^{FAK}$ (Figure 4, lanes A and B) but undetectable with PO-conjugated anti-pTyr antibody (Figure 5a and c), suggesting it to be a non-specific band detected by secondary antibody. To confirm tyrosine phosphorylation of $p125^{FAK}$, we immunoprecipitated $p125^{FAK}$ repeatedly from the same cancer tissue lysates and probed with anti-pTyr antibody together with whole cell lysate and supernatant (Figure 5). PO-conjugated anti-pTyr antibody was used in the following experiments to avoid non-specific binding of secondary antibody. Tyrosine phosphorylation of $p125^{FAK}$, which co-migrated with one of the two major pTyr-containing proteins was again clearly detected in tumour tissues of both cases and decreased by repeated immunoprecipitation (Figure 5a and c). Although repeated immunoprecipitation removed most of the tyrosine-phosphorylated $p125^{FAK}$ from the lysate (Figure 5a and b), the major pTyr-containing protein co-migrated with $p125^{FAK}$ remained in the supernatant fraction, suggesting that there may be additional pTyr-containing proteins of similar molecular size to $p125^{FAK}$.

To characterise other pTyr-containing proteins in cancer tissues, we next immunoprecipitated p120, α- and β-catenins, $p130^{Cas}$ or vinculin. These proteins are well-known tyrosine-phosphorylated proteins and have molecular sizes similar to 100–130 kDa phosphorylated proteins (Reynolds *et al.*, 1989; Hamaguchi *et al.*, 1993*a*; Matsuyoshi *et al.*, 1992; Mayer *et al.*, 1988; Sakai *et al.*, 1994; Sefton *et al.*, 1981). Two tyrosine-phosphorylated protein bands were immuno-

**Figure 4** Detection of p125$^{FAK}$ phosphorylation in surgical specimens. p125$^{FAK}$ in lung cancer was immunoprecipitated with anti-125$^{FAK}$ antibody and probed with anti-pTyr antibody as described in Materials and methods. A pair of normal (A) and cancer tissue (B) was immunoprecipitated without anti-p125$^{FAK}$ as a negative control. Normal (C and E) and cancer tissue (D and F) obtained from cases 2 (C and D) and 9 (E and F) was immunoprecipitated with anti-p125$^{FAK}$ antibody. (G) p125$^{FAK}$ from QG56 cells as a control.

**Figure 5** Tyrosine phosphorylation of p125$^{FAK}$ and p120 in surgical specimens. Lysates of cancer tissues were repeatedly immunoprecipitated with anti-p125$^{FAK}$ (**a**, **b** and **c**) or anti-p120 (**d** and **e**) and subsequently probed with anti-pTyr (**a**, **c** and **d**), anti-p125$^{FAK}$ (**b**) or anti-p120 (**e**). **a**, **b**, **d** and **e**, case 2; **c**, case 9. Lane 1, whole cell lysate; lane 2, first immunoprecipitated sample; lane 3, second immunoprecipitated sample; lane 4, supernatant of immunoprecipitation.

precipitated with anti-p120 antibody (Figure 5d and e), suggesting p120 as one of the major pTyr-containing proteins in cancer tissue. Tyrosine phosphorylation of α- and β-catenins was examined. Expression of α-catenin was low and its tyrosine phosphorylation was undetectable (Figure 6a and b). β-catenin was clearly tyrosine phosphorylated and co-migrated with a faint band below the two major pTyr-containing proteins of cancer tissues (Figure 6c and d). A pTyr-containing protein slightly larger than β-catenin was co-immunoprecipitated with β-catenin (Figure 6c). We could not identify protein species of this band. In addiiton to the results with case 2, we found tyrosine phosphorylation of p120 and β-catenin in another case (case 9). Tyrosine phosphorylation of vinculin and p130$^{Cas}$ was next examined. We could not detect phosphorylation of vinculin (Figure 7a and b). Since anti-p130$^{Cas}$ antibody was available only for immunoblotting, we immunoprecipitated pTyr-containing proteins from tissue lysate by anti-pTyr antibody and subsequently probed with anti-p130$^{Cas}$ antibody together with whole cell lysate and supernatant (Figure 7c and d). Although a faint band migrating slower than p130$^{Cas}$ was detected in immunoprecipitated fraction (Figure 7d, lane 2*), most of p130$^{Cas}$ remained in the supernatant (Figure 7d, lane 3), suggesting p130$^{Cas}$ is not the major pTyr-containing protein of lung cancer tissue.

We examined expression of EGF receptor and c-erbB-2 protein in these cancer tissues. All tumour tissues examined expressed EGF receptor to similar levels, but only two cases expressed c-erbB-2 in a tumour-specific manner and these cases were both adenocarcinoma with mediastinal node metastasis (data not shown).

### *Association of 100–130 kDa proteins' phosphorylation with clinicopathological manifestations*

Tables I and II are the summary of clinicopathological manifestations in the cases we examined. These objectives consisted of 19 squamous cell, 23 adeno, 1 adenosquamous

**Figure 6** Tyrosine phosphorylation of catenins in surgical specimens. Lysates of cancer tissues (case 2) were repeatedly immunoprecipitated with anti-α-catenin (**a** and **b**) or anti-β-catenin (**c** and **d**) and probed with anti-pTyr (**a** and **c**) anti-α-catenin (**b**) or anti-β-catenin (**d**). Lane 1, whole cell lysate; lane 2, first immunoprecipitated sample; lane 3, second immunoprecipitated sample; lane 4, supernatant of immunoprecipitation.

**Figure 7** Detection of vinculin and $p130^{Cas}$ in surgical specimens. Lysates of cancer tissues (case 2) were immunoprecipitated with anti-vinculin (**a** and **b**) or anti-pTyr (**c** and **d**) antibody and probed with anti-pTyr (**a** and **c**), anti-vinculin (**b**) or anti-$p130^{Cas}$ (**d**) antibody. Lane 1, whole cell lysate; lane 2, first immunoprecipitated sample; lane 3, in **a**, second immunoprecipitated sample; lane 3, in **c** and **d**, supernatants; lane 4 in **a**, supernatant of immunoprecipitation.

and 1 small-cell carcinoma, and were obtained from 33 male and 11 female patients with an age range of 49 to 78 years (average 65 years). We found that 20 cancer tissues (45% of those examined) showed tumour-specific phosphorylation of 100–130 kDa proteins (Table I). Tyrosine phosphorylation of these proteins was found in 47% (9 of 19 cases) of squamous cell carcinoma tissues and 43% (10 of 23 cases) of adenocarcinoma tissues. Tyrosine phosphorylation of these proteins was not associated with age ($P=0.54$), sex ($P=0.29$), the histological classification ($P=0.55$), the tumour size ($P=0.95$), or pathological T factor of TNM classification ($P=0.10$) (Table I). However, we found that the cases with nodal involvement (N1 and N2) had clearly higher incidence of tyrosine phosphorylation of these proteins compared with those with no nodal involvement (N0) ($P=0.01$) (Table I). In addition, cases which showed tyrosine phosphorylation had a shorter survival length after operation compared with those without tyrosine phosphorylation ($P=0.01$). To extend these observations, we next examined the relationship between clinicopathological variables and disease-free survival time (Table II). Because of short post-operative follow-up time, most of the factors did not affect survival time of patients except for the protein phosphorylation of 100–130 kDa proteins ($P=0.01$) and p-N factor ($P=0.03$). Surprisingly, three of eight pathological T1N0 cases phosphorylating 100–130 kDa proteins relapsed in one year after operation. To confirm further, correlation of disease-free survival time with phosphorylation of 100–130 kDa proteins and nodal involvement was examined by Cox's proportional hazard model. Correlation of survival length with tyrosine phos-

Table I Tyrosine phosphorylation of 100–130 kDa proteins and clinicopathological variables for patients with human lung cancer

| | Phosphorylation of 100–130 kDa proteins Negative (n=24) | Positive (n=20) | P-value[a] |
|---|---|---|---|
| Male | 16 | 17 | |
| Female | 8 | 3 | 0.29 |
| Primary tumour | | | |
| 1 | 8 | 1 | |
| 2 | 9 | 10 | |
| 3 | 6 | 6 | 0.10 |
| 4 | 1 | 3 | |
| Regional lymph nodes | | | |
| 0 | 22 | 8 | |
| 1 | 1 | 5 | 0.001 |
| 2 | 1 | 7 | |
| Metastasis | | | |
| 0 | 24 | 20 | |
| 1 | 0 | 0 | – |
| Surgical stage | | | |
| 1 | 17 | 6 | |
| 2 | 0 | 4 | |
| 3A | 6 | 7 | 0.02 |
| 3B | 1 | 3 | |
| Histology | | | |
| Adenocarcinoma | 13 | 10 | |
| Squamous cell carcinoma | 10 | 9 | |
| Adenosquamous carcinoma | 1 | 0 | 0.55 |
| Small-cell carcinoma | 0 | 1 | |
| Continuous variables | | | |
| Age (years) | 65.4 ± 7.7[b] | 64.3 ± 7.4 | 0.54 |
| Tumour size (cm) | 3.8 ± 1.9 | 3.7 ± 1.6 | 0.95 |
| Survival (days) | 928.0 ± 74.6 | 316.8 ± 33.8 | 0.01 |

[a] P-values calculated using Fisher's exact test and Mann–Whitney U-test. The value for survival was calculated using the log-rank test as determined by Kaplan and Meier. [b] Mean ± s.d.

phorylation of 100–130 kDa proteins was statistically significant ($P=0.03$), although survival length showed poor correlation with nodal involvement ($P=0.16$). The cumulative probability of disease-free survival is displayed in Figure 8 for p-stage of TNM and Figure 9 for 100–130 kDa proteins' phosphorylation. These results strongly suggested that the tyrosine phosphorylation of 100–130 kDa proteins, probably including $p125^{FAK}$, p120 and β-catenin, correlates with a malignant phenotype of lung cancer.

### Discussion

Evidence has been accumulated that oncogenes that encode tyrosine kinases are involved in tumorigenesis of human cancer. The results presented in this report demonstrate that, in lung cancer tissues, a subset of 100–130 kDa proteins was indeed tyrosine phosphorylated in a tumour-specific manner. Of 44 cases of lung cancer we examined, 20 cases showed tyrosine phosphorylation of these proteins. We found that the profiles of phosphorylation in cancer tissues resembled each other and similar phosphorylated proteins were also found in oesophageal and colorectal cancer tissues, despite the difference in histological types and origins of cancer. In addition, statistical analysis showed good correlation of these protein phosphorylations with poor prognosis of patients. These results suggest that signalling via tyrosine phosphorylation of these proteins may play an important role in tumorigenesis.

By histochemical analysis, amplification of EGF receptor (Cerny *et al.*, 1986; Veale *et al.*, 1987; Dazzi *et al.*, 1989; Berger *et al.*, 1987; Haeder *et al.*, 1989; Siegfried, 1987) and c-erbB-2 (Kern *et al.*, 1990; Schneider *et al.*, 1989; Weiner *et al.*, 1990) in human lung cancer tissues has been reported. However, we found no clear correlation between the levels of EGF receptor of c-erbB-2 expression and the levels of tyrosine phosphorylation in cancer tissues (data not shown). Moreover, neither tyrosine-phosphorylated proteins corresponding to autophosphorylated EGF receptor nor that of c-erbB-2 protein were found in cancer tissues. These results suggest that other kinases in addition to EGF receptor and c-erbB-2 may be required for the phosphorylation of 100–130 kDa proteins.

Table II Disease-free survival and clinicopathological variables for patients with human lung cancer

| | Survival (days) | P-value[a] |
|---|---|---|
| Sex | | |
| Male (31) | 780.6 ± 79.8[b] | |
| Female (10) | 346.7 ± 48.3 | 0.50 |
| Primary tumour | | |
| 1 (8) | 1069.0 ± 0 | |
| 2 (17) | 369.4 ± 34.0 | 0.07 |
| 3 (12) | 370.5 ± 29.6 | |
| 4 (4) | 408.0 ± 193.5 | |
| Regional lymph nodes | | |
| 0 (28) | 844.2 ± 79.9 | |
| 1 (6) | 736.2 ± 182.3 | 0.002 |
| 2 (7) | 244.7 ± 42.6 | |
| Surgical stage | | |
| 1 (21) | 818.1 ± 95.3 | |
| 2 (4) | 313.0 ± 90.1 | |
| 3A (12) | 370.5 ± 29.6 | 0.16 |
| 3B (4) | 408.0 ± 193.5 | |
| Histology | | |
| Adenocarcinoma (21) | 550.3 ± 107.4 | |
| Squamous cell carcinoma (18) | 899.8 ± 82.0 | |
| Adenosquamous carcinoma (1) | 368.0 ± 0 | 0.11 |
| Small-cell carcinoma (1) | 359.0 ± 0 | |
| 100–130 kDa proteins phosphorylation | | |
| Negative (22) | 928.0 ± 74.6 | |
| Positive (19) | 316.8 ± 33.8 | 0.01 |

[a] P-value for disease-free survival was calculated using the log-rank test as determined by Kaplan and Meier. [b] Mean ± s.d.

**Figure 8** Disease-free survival curve of all patients according to pathological stage. The Kaplan–Meier method was used to estimate the survival distribution for each subgroup. The log-rank test was used to evaluate the equality of the survival curves.

**Figure 9** Disease-free survival curve of all patients with or without tyrosine phosphorylation of 100–130 kDa proteins. The Kaplan–Meier method was used to estimate the survival distribution for each subgroup. The log-rank test was used to evaluate the equality of the survival curves.

By immunoprecipitation with specific antibodies, we found tyrosine phosphorylation of p125$^{FAK}$, p120 and β-catenin in lung cancer tissues. Attention has been drawn to p125$^{FAK}$ by its unique structure and character (Schaller *et al.*, 1992). This kinase was found as a major pTyr-containing protein in RSV-transformed cells and appeared to localise to focal adhesions. Recent studies suggest that p125$^{FAK}$ kinase is involved in multifarious cellular functions such as cell adhesion (Kornberg *et al.*, 1992), shape (Burridge *et al.*, 1992), motility and growth (Zachary *et al.*, 1992) as well as transformation. Targeting of p125$^{FAK}$ gene in mice resulted in a defect of mesoderm development, and cells from these embryos had reduced mobility *in vitro* (Illic *et al.*, 1995). p125$^{FAK}$ becomes tyrosine phosphorylated and activated in response to integrin-mediated binding of cells to the extracellular matrix, suggesting p125$^{FAK}$ phosphorylation is important for cell adhesion and/or migration (Kornberg *et al.*, 1992; Burridge *et al.*, 1992). β-catenin is one of the components of the cadherin–catenin cell adhesion system and appears to play a crucial role in cadherin-dependent cell adhesion. In the human gastric cancer cell line, HSC-39, mutation in the β-catenin gene that resulted in complete abolishment of E-cadherin-dependent cell–cell adhesion was observed, and transfection of the β-catenin gene in the cells fully recovered the cell–cell adhesion (Kawanishi *et al.*, 1995). We have previously reported (Matsuyoshi *et al.*, 1992; Hamaguchi *et al.*, 1993*a*) that cadherin dependent cell–cell adhesion was strongly perturbed upon tyrosine phosphorylation of β-catenin in RSV-transformed cells where cadherins and catenins were expressed and formed complexes as normal cells. Another pTyr-containing protein, p120, was also identified as a major pTyr-containing protein in RSV-transformed cells whose phosphorylation closely correlated with cell transformation (Reynolds *et al.*, 1989). Later study showed that p120 had close homology with a group of cell adhesion molecules, β-catenin, plakoglobin and *armadillo* (Reynolds *et al.*, 1992), suggesting that p120 may also play a role in cell adhesion. Thus, tyrosine phosphorylation of these adhesion molecules may result in suppression of cell–cell adhesion and activated lung cancer cell migration, invasion and metastasis.

Besides p125$^{FAK}$, 120 and β-catenin, other tyrosine-phosphorylated proteins ranged 100–130 kDa were found in cancer tissue. At present, we do not know precisely how many and what kind of proteins are included. These tyrosine-phosphorylated proteins included neither p130$^{Cas}$, α-catenin nor vinculin. We found, however, that this subset of tyrosine-phosphorylated proteins was found in various types of human cancer cell lines (Figure 1) and in the cancer tissues of other organs (Figure 3). Further characterisation of the species of the tyrosine-phosphorylated proteins is an important problem to be studied.

#### Acknowledgements

We are grateful to Dr H Hanafusa for his continuous encouragement and support. This work was supported in part by a grant-in-aid for cancer research from the Ministry of Education, Science and Culture, Japan, a grant from the Sankyo Foundation of Life Science, and a grant from the Foundation for Promotion of Cancer Research.

### References

BERGER MS, GULLICK WJ, GREENFIELD C, EVANS S, ADDIS BJ AND WATERFIELD MD. (1987). Epidermal growth factor receptors in lung tumours. *J. Pathol.*, **152,** 297–307.

BURRIDGE K, TURNER CE AND ROMER LH. (1992). Tyrosine phosphorylation of paxillin and pp125$^{FAK}$ accompanies cell adhesion to extracellular matrix: a role in cytoskeletal assembly. *J. Cell. Biol.*, **119,** 893–903.

CERNY T, BARNES DM, HASLETON P, BARBER PV, HEALY K, GULLICK W AND THATCHER N. (1986). Expression of epidermal growth factor receptor (EGF-R) in human lung tumours. *Br. J. Cancer*, **54,** 265–269.

DAZZI H, HASLETON PS, THATCHER N, BARNES DM, WILKES S, SWINDELL R AND LAWSON RAM. (1989). Expression of epidermal growth factor receptor (EGF-R) in non-small-cell lung cancer. Use of archival tissue and correlation of EGF-R with histology, tumour size, node status and survival. *Br. J. Cancer*, **59,** 746–749.

HAEDER M, ROTSCH M, BEPLER G, HENNING C, HAVEMANN K, HEIMANN B AND MOELLING K. (1988). Epidermal growth factor receptor expression in human lung cancer cell lines. *Cancer Res.*, **48,** 1132–1136.

HAMAGUCHI M, GRANDORI C AND HANAFUSA H. (1988). Phosphorylation of cellular proteins in Rous sarcoma virus-infected cells: analysis by use of anti-pTyr antibodies. *Mol. Cell. Biol.*, **8,** 3035–3042.

HAMAGUCHI M, MATSUYOSHI N, OHNISHI Y, GOTOH B, TAKEICHI M AND NAGAI Y. (1993*a*). p60$^{v\text{-}src}$ causes tyrosine phosphorylation and inactivation of the N-cadherin-catenin cell adhesion system. *EMBO J.*, **12,** 559–564.

HAMAGUCHI M, XIAO H, UEHARA Y, OHNISHI Y AND NAGAI Y. (1993*b*). Herbimycin A inhibits the association of p60$^{v\text{-}src}$ with the cytoskeletal structure and with phosphatidylinositol 3′ kinase. *Oncogene*, **8,** 559–564.

ILIC D, FURUTA Y, KANAZAWA A, TAKEDA N, SOBUE K, NAKATSUJI N, NOMURA S, FUJIMOTO J, OKADA M, YAMAMOTO T AND AIZAWA S. (1995). Reduced cell motility and enhanced focal adhesion contact formation in cells from FAK-deficient mice. *Nature*, **377**, 539–544.

KAWANISHI J, KATO J, SASAKI K, FUJII S, WATANABE N AND NIITSU Y. (1995). Loss of E-cadherin-dependent cell–cell adhesion due to mutation of the β-catenin gene in a human cancer cell line, HSC-39. *Mol. Cell. Biol.*, **15**, 1175–1181.

KERN JA, SCHWARTZ DA, NORDBERG JE, WEINER DB, GREENE MI, TORNEY L AND ROBINSON RA. (1990). p185$^{neu}$ expression in human lung adenocarcinomas predicts shortened survival. *Cancer Res.*, **50**, 5184–5191.

KINJO M, OKA K, KOHGA S, TANAKA K, OBOSHI S, HAYATA Y AND YASUMOTO K. (1979). Thromboplastic and fibrinolytic activities of cultured human cancer cell lines. *Br. J. Cancer*, **39**, 15–23.

KORNBERG L, EARP HS, PARSONS JT, SCHALLER M AND JULIANO RL. (1992). Cell adhesion or integrin clustering increases phosphorylation of a focal adhesion-associated tyrosine kinase. *J. Biol. Chem.*, **267**, 23439–23442.

MACAULAY VM, TEALE JD, EVERARD MJ, JOSHI GP, SMITH IE AND MILLAR JL. (1988). Somatomedin-C/insulin-like growth factor-I is a mitogen for human small-cell lung cancer. *Br. J. Cancer*, **57**, 91–93.

MACAULAY VM, EVERARD MJ, TEALE JD, TROTT PA, VAN WYK JJ, SMITH IE AND MILLAR JL. (1990). Autocrine function for insulin-like growth factor I in human small cell lung cancer cell lines and fresh tumour cells. *Cancer Res.*, **50**, 2511–2517.

MATSUYOSHI N, HAMAGUCHI M, NAGAFUCHI A, TSUKITA S, TANIGUCH S AND TAKEICHI M. (1992) Cadherin-mediated cell–cell adhesion is perturbed by v-*src* tyrosine phosphrylation in metastatic fibroblasts. *J. Cell Biol.*, **118**, 703–714.

MAYER BJ, HAMAGUICHI M AND HANAFUSA H. (1988). A novel viral oncogene with structural similarity to phospholipase C. *Nature*, **332**, 272–275.

REYNOLDS AB, ROESEL DJ, KANNER SB AND PARSONS JT. (1989). Transformation-specific tyrosine phosphorylation of a novel cellular protein in chicken cells expressing oncogenic variants of the avian cellular *src* gene. *Mol. Cell. Biol.*, **9**, 629–938.

REYNOLDS AB, HERBERT L, CLEVELAND JL, BERG ST AND GAUT JR. (1992). p120, a novel substrate of protein tyrosine kinase receptors and of p60$^{v\text{-}src}$ is related to cadherin-binding factors β-catenin, plakoglobin and *armidillo*. *Oncogene*, **7**, 2439–2445.

SAGA S, HAMAGUCHI M, HOSHINO M AND KOJIMA K. (1985). Expression of meta-vinculin associated with differentiation of chicken embryonal muscle cells. *Exp. Cell Res.*, **156**, 45–56.

SAKAI R, IWAMATSU A, HIRANO N, OGAWA S, TANAKA T, MANO H, YAZAKI Y AND HIRAI H. (1994). A novel signalling molecule, p130, forms stable complexes *in vivo* with v-Crk and v-Src in a tyrosine phosphorylation-dependent manner. *EMBO J.*, **13**, 3748–3756.

SCHALLER MD, BORGMAN CA, COBB BS, VINES RR, REYNOLDS AB AND PARSONS JT. (1992). pp125$^{FAK}$, a structurally distinctive protein-tyrosine kinase associated with focal adhesions. *Proc. Natl Acad. Sci. USA*, **89**, 5192–5196.

SCHNEIDER PH, HUNG M-C, CHIOKKA S, MANNING J, ZHAO X, FANG K AND ROTH JA. (1989). Differential expression of the c-*erb*B-2 gene in human small-cell and non small-cell lung cancer. *Cancer Res.*, **49**, 4968–4971.

SEFTON BM, HUNTER T, BALL EH AND SINGER SJ. (1981). Viculin: a cytoskeletal target of the transforming protein of Rous sarcoma virus. *Cell*, **13**, 751–760.

SIEGFRIED JM. (1987). Detection of human lung epithelial cell growth factors produced by a lung carcinoma cell line: use in culture of primary solid lung tumours. *Cancer Res.*, **47**, 2903–2910.

TAKESHIMA E, HAMAGUCHI M, WATANABE T, AKIYAMA S, KATAOKA M, OHNISHI Y, XIAO H, NAGAI Y AND TAKAGI H. (1991). Aberrant elevation of tyrosine-specific phosphorylation in human gastric cancer cells. *Jpn. J. Cancer Res.*, **82**, 1428–1435.

VEALE D, ASHCROFT T, MARSH C, GIBSON GJ AND HARRIS AL. (1987). Epidermal growth factor receptors in non-small-cell lung cancer. *Br. J. Cancer*, **55**, 513–516.

WEINER DB, NORDBERG J, ROBINSON R, NOWEL PC, GAZDAR A, GREENE MI, WILLIAMS WV, COHEN JA AND KERN JA. (1990). Expression of the neu gene-encoded protein (P185$^{neu}$) in human non-small cell carcinomas of the lung. *Cancer Res.*, **50**, 421–425.

ZACHARY I, SMITH JS AND ROZENGURT E. (1992). Bombesin, vasopressin, and endothelin stimulation of tyrosine phosphorylation in Swiss 3T3 cells. *J. Biol. Chem.*, **267**, 19031–19034.